# Automated Landmark Annotation for Morphometric Analysis of Distal Femur and Proximal Tibia

**DOI:** 10.3390/jimaging10040090

**Published:** 2024-04-11

**Authors:** Jonas Grammens, Annemieke Van Haver, Imelda Lumban-Gaol, Femke Danckaers, Peter Verdonk, Jan Sijbers

**Affiliations:** 1 Antwerp Surgical Training, Anatomy and Research Centre (ASTARC), University of Antwerp, Wilrijk, 2610 Antwerp, Belgium; 2 imec-VisionLab, Department of Physics, University of Antwerp, Wilrijk, 2610 Antwerp, Belgium; femke.danckaers@uantwerpen.be (F.D.);; 3 MoRe Institute, Deurne, 2100 Antwerp, Belgium; 4 Nicolaas Institute of Constructive Orthopaedic Research and Education Foundation for Arthroplasty and Sports Medicine, Medistra Hospital, Jakarta 12950, Indonesia; pina_mail2000@yahoo.com; 5 OrthoCA (Orthopaedic Center Antwerp), Deurne, 2100 Antwerp, Belgium; 6 Department of Orthopaedics, University Hospitals Antwerp, Edegem, 2650 Antwerp, Belgium

**Keywords:** morphometrics, 3D landmark analysis, knee MRI, orthopedics

## Abstract

Manual anatomical landmarking for morphometric knee bone characterization in orthopedics is highly time-consuming and shows high operator variability. Therefore, automation could be a substantial improvement for diagnostics and personalized treatments relying on landmark-based methods. Applications include implant sizing and planning, meniscal allograft sizing, and morphological risk factor assessment. For twenty MRI-based 3D bone and cartilage models, anatomical landmarks were manually applied by three experts, and morphometric measurements for 3D characterization of the distal femur and proximal tibia were calculated from all observations. One expert performed the landmark annotations three times. Intra- and inter-observer variations were assessed for landmark position and measurements. The mean of the three expert annotations served as the ground truth. Next, automated landmark annotation was performed by elastic deformation of a template shape, followed by landmark optimization at extreme positions (highest/lowest/most medial/lateral point). The results of our automated annotation method were compared with ground truth, and percentages of landmarks and measurements adhering to different tolerances were calculated. Reliability was evaluated by the intraclass correlation coefficient (ICC). For the manual annotations, the inter-observer absolute difference was 1.53 ± 1.22 mm (mean ± SD) for the landmark positions and 0.56 ± 0.55 mm (mean ± SD) for the morphometric measurements. Automated versus manual landmark extraction differed by an average of 2.05 mm. The automated measurements demonstrated an absolute difference of 0.78 ± 0.60 mm (mean ± SD) from their manual counterparts. Overall, 92% of the automated landmarks were within 4 mm of the expert mean position, and 95% of all morphometric measurements were within 2 mm of the expert mean measurements. The ICC (manual versus automated) for automated morphometric measurements was between 0.926 and 1. Manual annotations required on average 18 min of operator interaction time, while automated annotations only needed 7 min of operator-independent computing time. Considering the time consumption and variability among observers, there is a clear need for a more efficient, standardized, and operator-independent algorithm. Our automated method demonstrated excellent accuracy and reliability for landmark positioning and morphometric measurements. Above all, this automated method will lead to a faster, scalable, and operator-independent morphometric analysis of the knee.

## 1. Introduction

In orthopedic clinical practice, patient-specific instruments and implants are highly dependent on accurate anatomical characterization for the region of interest, for which landmark analysis is a commonly used technique. It involves the use of 3D coordinates of anatomically meaningful points to calculate relative distances and angles.

Several knee-specific applications exist, including a sizing and positioning tool for joint replacement surgery [1], a matching tool for donors and acceptors in meniscal allograft transplantation [2], or large-scale morphometric risk factor analysis [3,4,5]. All these applications are based on a set of anatomical landmarks at the level of the distal femur and proximal tibia. Traditional methods to identify anatomical landmarks involve manual annotation, which is highly time-consuming and prone to substantial intra- and inter-observer variability [6,7]. Time consumption depends mainly on image quality and experience level, plateauing towards the end of the learning curve. Clear landmark definitions and well-described protocols are essential to limit this human operator-induced variability [8].

Various automated approaches have been developed to minimize subjectivity and processing time in annotation tasks. They can be divided into three large groups: knowledge-based, template-based and learning-based [9]. The first category exploits knowledge of the human anatomy to characterize landmarks by a set of unique geometric or anatomical characteristics such as surface normal direction, local curvature, or spatial relation to other landmarks [10]. Template-based approaches like statistical shape models start with an annotated template image or template mesh, which is elastically deformed to match the image or mesh of the subject’s anatomy [11]. Lastly, several deep learning-based approaches have been proposed [12,13], mainly consisting of combinations or modifications to convolutional neural networks.

We propose a hybrid methodology combining a template-based algorithm with knowledge-based optimization of landmark positions. More specifically, a fully automated landmark annotation method is proposed for measurement of the medial and lateral tibiofemoral joints in all three directions. Anatomical correspondences are established by 3D surface registration [11], after which the landmark seeds are propagated from the template mesh to the subject’s 3D bone and cartilage surface meshes. Finally, landmarks defined at local extreme positions were optimized to ensure their position at that outermost point.

Our proposed automated landmarking tool is compared with the average landmark coordinates and measurements from three independent expert observers. Furthermore, the landmarking tool is tested for the 3D morphometric analysis of the tibiofemoral joint, based on a retrospectively collected dataset of 3D models of 20 knees. It does so by comparing the intra- and inter-observer variability (manual, within, and between observers) with the inter-method (manual versus automated method) variability and reliability. We hypothesize the inter-method landmark variability to be non-inferior to the inter-observer landmark positioning variability. Furthermore, we hypothesize that our automated morphometric measurement variability is in line with the inter-observer morphometric measurement variability.

## 2. Methods

### 2.1. Data and Workflow

From a large multicenter database of arthroscopic partial meniscectomy patients, 20 randomly selected anonymized pre-operative knee MRI scans were randomly selected: 15 male (mean age ± SD: 49 ± 13 years) and 5 female (mean age ± SD: 58 ± 13 years) subjects.

Subjects with osteophytes (OARSI grade I–III) or major knee deformities (>5° varus or valgus by clinical judgement) were excluded from the study. Informed consent was obtained from all participants prior to their inclusion and the study protocol was approved by the ethical committees according to the 1964 Declaration of Helsinki and its later amendments.

#### 2.1.1. Manual Segmentation

The MRI scan data were loaded into Mimics 22.0 (Materialise, Leuven, Belgium) to create 3D models of the distal femur and proximal tibia. For all subjects, MRI image sets in the three perpendicular anatomical planes were available, following the standard clinical MRI protocols. At least two MRI image sets with a perpendicular acquisition plane were used to segment the distal femur and proximal tibia into two separate 3D models consisting of bone and cartilage. The resulting projected contours of the 3D models were then visually double-checked on the remaining available image sets and manually fine-tuned in an iterative workflow using the “Contour edit” tool of the software package. Finally, the 3D models of the distal femur and proximal tibia were saved as triangular 3D surface meshes.

#### 2.1.2. Manual Landmark Annotation

All included subjects (*n* = 20) were manually landmark-annotated by three trained observers: one junior researcher (JG), one senior researcher (AVH), and one orthopedic surgeon (ILG). All observers had at least 2 years of experience. One observer (JG) performed all landmark annotations three times in a random case order with a minimum interval of 1 month to avoid recall bias. The landmarks and morphometric measurements of interest are defined and further described in ‘Section 3’.

#### 2.1.3. Automated Landmark Annotation

##### Registration of 3D Bone and Cartilage Models

Three-dimensional surface registration was performed to obtain a dense set of anatomically corresponding pseudo-landmarks. The first step consisted of isotropic remeshing of an arbitrary chosen bone and cartilage shape of interest [14], which served as a template. Next, an iterative algorithm of rigid and constrained elastic deformations [11] was used to register all 3D bone and cartilage meshes with the template. In short, the template mesh was progressively deformed into the target mesh while adhering to specific geometric constraints (e.g., surface distance, surface normal direction) to preserve anatomical correspondence between the template and target. By doing so, the topology of the template mesh was imposed on each of the target meshes. The resulting deformed meshes could then be used as a replacement for the original meshes. Next, the mean distal femur and proximal tibia bone and cartilage shape were calculated by averaging the corresponding point coordinates of the deformed meshes. Using the same iterative registration process as described before, all 20 bone and cartilage shapes were again registered with the mean bone and cartilage shape as a template. The mean distal femur and proximal tibia meshes consisted of 47,622 and 46,721 vertices, respectively. All resulting deformed meshes were visually checked for resulting mesh quality (triangle distortion).

##### Landmark Propagation

**Figure 1:**
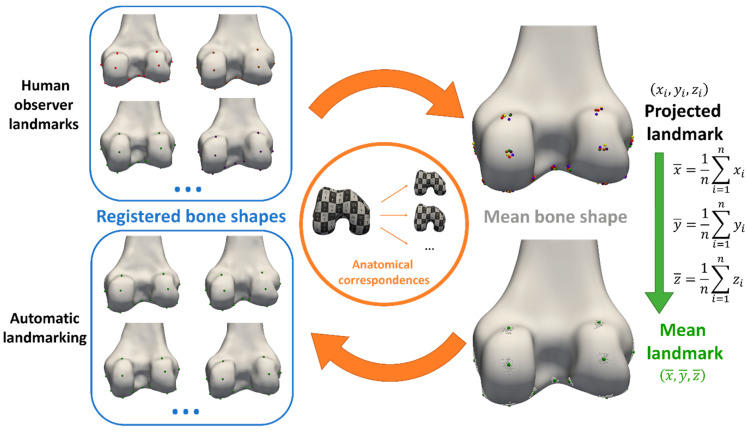
Workflow overview. Landmarks were annotated manually by three experts (**top left**) and propagated to the mean bone and cartilage shape (**top right**), leaving out the observations of the subject for automated landmarking. Next, mean landmark coordinates were calculated from all propagated observations on the mean bone and cartilage shape (**bottom right**). Finally, automated landmarking initialization was performed by propagating the landmarks back to the subject’s bone and cartilage shapes, using the previously established anatomical correspondences again (**bottom left**).

From the obtained anatomical correspondences, all manually annotated landmarks (cf. Section 2.1.2) of all but one subject (the subject of interest for automated landmark annotation) were propagated to the mean bone and cartilage shape in a leave-one-out experiment. Their coordinates were averaged to define the mean landmark position, projected on the mean bone and cartilage shape. To ensure equal weighting of the three observers in the mean of the landmark projections, the three landmark projections of observer 1 were averaged beforehand. Next, automated landmark annotation of all subjects was initialized by propagating the mean landmark positions to the corresponding vertices of the subjects. In a final step, the landmark positions at extreme locations were optimized according to their definition (Section 3.1) via a custom Python script (and VTK library [15]) to ensure their position was the most anterior/posterior, medial/lateral or proximal/distal point. The complete workflow is summarized in Figure 1.

## 3. Observations

### 3.1. Landmark Definitions

**Figure 2:**
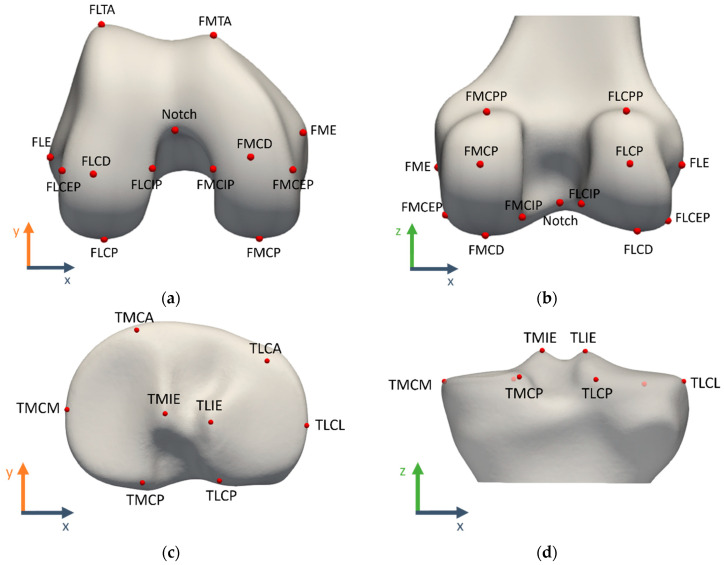
Landmark visualization on the template bone and cartilage shapes: (**a**) distal femoral view, (**b**) posterior femoral view, (**c**) proximal tibial view, (**d**) posterior tibial view. A complete overview of landmark definitions can be found in Table 1.

The landmark definitions were adopted from earlier reported landmark studies [3,5,6,7]. The landmarks were manually annotated in 3D Slicer, first directly on the 3D model and eventually fine-tuned on the relevant MRI view with projected 3D model contours. A complete overview of the evaluated landmarks with their acronyms and definitions is given in Table 1 and illustrated in Figure 2.

#### 3.1.1. Reference Coordinate System Definition

Clinical MRI scans are characterized by a certain field of view, limited to the distal femur and proximal tibia. It is assumed that all knees were correctly positioned by the MRI operators: horizontal and straight on the MRI patient table, leaving only one degree of freedom for minor internal or external rotation of the hip during image acquisition. Therefore, all 3D models of the femur and tibia were rotated in the axial plane to make the femoral posterior condylar line parallel to the mediolateral x-axis.

The reference coordinate system is thus defined by the following axes:

- x-axis (mediolateral): parallel to the femoral posterior condylar line, defined by the FMCP and FLCP landmarks (mean of 3 observers as ground truth)
- y-axis (anteroposterior): common perpendicular to the x- and z-axes
- z-axis (proximodistal): MRI patient table movement direction

#### 3.1.2. Morphometric Measurement Definitions

**Figure 3:**
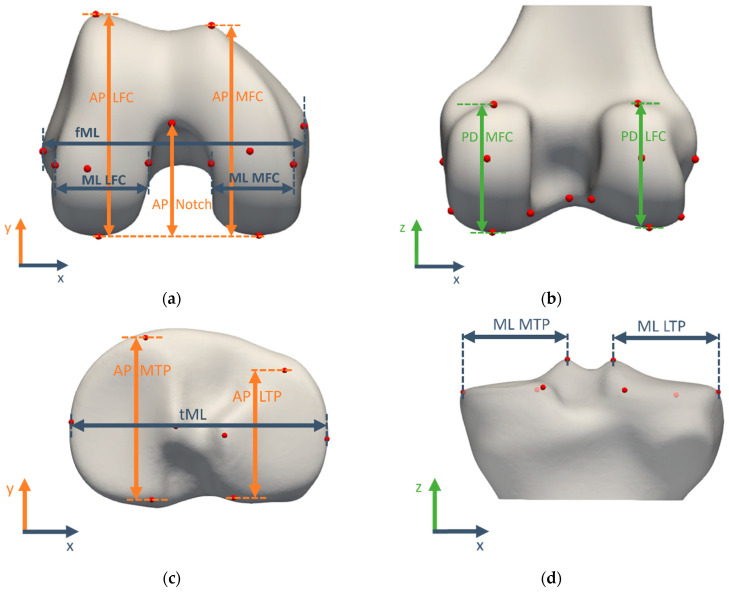
Morphometric measurements visualized on the template bone and cartilage shapes: (**a**) distal femoral view, (**b**) posterior femoral view, (**c**) proximal tibial view, (**d**) posterior tibial view. A complete overview of morphometric measurement definitions can be found in Table 2.

The morphometric measurements for validation were summarized in Table 2 and visualized in Figure 3. They characterize the size of the medial and lateral compartments of both the femur and tibia in all clinically relevant directions by projecting the landmarks of interest to the relevant axis.

### 3.2. Validation Study for Manual Morphometric Analysis

#### 3.2.1. Landmark Validation

Manual landmark annotations were considered the gold standard. For the intra-observer error assessment, ground truth landmarks were defined as the average landmark coordinates over three observations of observer 1 and were also used as final landmark annotations for observer 1. For the inter-observer assessment, the ground truth landmarks were defined as the average landmark coordinates over the three observers and further referred to as the expert mean landmarks.

#### 3.2.2. Measurement Validation

Alignment to the reference coordinate system was performed using the expert mean landmarks. All measurements were calculated according to their definition in the previous section. For intra-observer variability assessment, the mean of three measurements by observer 1 served as the ground truth whereas for inter-observer variability evaluation, the mean measurement from three different observers served as the ground truth and is further referred to as the expert mean measurement. Absolute differences with the ground truth were calculated as a measure of variability.

### 3.3. Validation Study for Automated Morphometric Analysis

#### 3.3.1. Automated Landmark Validation

Expert mean landmarks served as ground truth landmark positions. Euclidean distances between the automatically determined landmarks and the ground truth landmarks were calculated as a measure of inter-method landmark variability.

#### 3.3.2. Automated Measurement Validation

Expert mean measurements served as ground truth morphometric measurements. Automated measurements were calculated from the automated landmark coordinates. Absolute differences were used as a measure of inter-method measurement variability.

### 3.4. Time Consumption

The required time to annotate all femoral and tibial landmarks was tracked for one observer and five subject cases. The time needed for the surface registration, extraction of the landmark positions and measurement calculations was derived from the filesystem metadata.

### 3.5. Statistical Analysis

All statistical analyses were performed in R 4.2.1, using the ‘irr’ package for ICC calculations [16,17].

#### 3.5.1. Landmark Positions

The first quartile (Q_1_), median (Q_2_), and third quartile (Q_3_) of the Euclidean distance to the ground truth landmark position were calculated per landmark for intra-observer, inter-observer, and inter-method variability. The interquartile range (IQR = Q_3_ − Q_1_) was used to define outliers. Observations below Q_1_ − 1.5 × IQR or above Q_3_ + 1.5 × IQR were considered outliers. Per knee scan, the number of outliers in the three experiments (intra-observer, inter-observer and manual versus automated landmark positioning differences) was calculated and reported in Table S1 in the Supplementary Materials for assessment of the robustness of the proposed method against bony anatomy variations. The mean and standard deviation of the difference between each observation and the ground truth are calculated per landmark.

#### 3.5.2. Measurements

Similarly, quartiles and medians were calculated per measurement for intra-observer, inter-observer, and inter-method variability. Outliers were again defined as observations that fall below Q_1_ − 1.5 × IQR or above Q_3_ + 1.5 × IQR. Per knee scan, the number of outliers in the three experiments (intra-observer, inter-observer and manual versus automated measurement differences) was calculated and reported in Table S2 in the Supplementary Materials for assessment of the robustness of the proposed method against bony anatomy variations. The mean and standard deviation of the absolute differences between each measurement and the mean measurement (intra-observer: three observations of observer 1; inter-observer: three observers) were calculated. Measurement reliability analysis included intra- and inter-observer reliability, reported as intraclass correlation coefficients (ICC) from two-way mixed (intra-observer) or two-way random (inter-observer) effects, absolute agreement, and single-rater models [18]. Values below 0.5, between 0.5 and 0.75, between 0.75 and 0.9, and above 0.9 were, respectively, considered to have poor, moderate, good, and excellent reliability. Inter-method (manual versus automated) agreement was also assessed by the ICC (two-way random effects, absolute agreement, single measurement). Finally, Bland–Altman plots visualized the inter-method (manual versus automated) agreement for all measurements [19].

## 4. Results

### 4.1. Validation Study for Manual Morphometric Analysis

#### 4.1.1. Manual Landmark Position Validation

**Figure 4:**
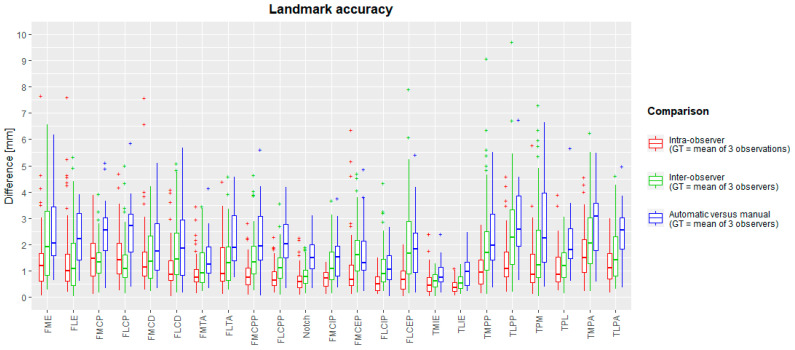
Box-and-whisker diagrams for intra-observer, inter-observer and inter-method landmark differences, calculated as Euclidean distances from the ground truth. The boxes indicate the IQR, the line within stands for the median and the whiskers indicate points < 1.5 IQR from the box. ‘+’ represents outliers. IQR: interquartile range, between first and third quartile. GT: ground truth.

The median landmark position intra-observer difference with the ground truth varied between 0.35 mm (TLIE) and 1.49 mm (TMPA). Maximal intra-observer landmark position differences were between 1.11 mm (TLIE) and 7.66 mm (FME). The median inter-observer difference was between 0.52 mm (TLIE) and 2.28 mm (TLPP). Maximal inter-observer landmark positions ranged between 1.24 mm (TLIE) and 9.7 mm (TLPP). The mean (and standard deviation) of inter-observer landmark differences was 1.53 (±1.22) mm. A complete overview is plotted in a box-and-whiskers diagram in Figure 4 (intra-observer: red; inter-observer: green).

#### 4.1.2. Manual Measurement Validation

**Figure 5:**
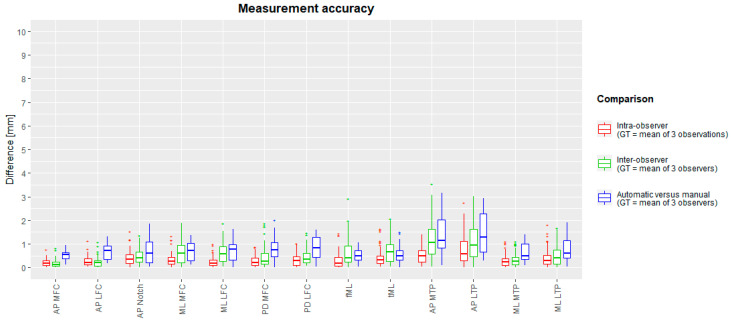
Box-and-whisker diagrams for intra-observer, inter-observer, and inter-method (manual versus automated) measurement differences. The mean measurement of three observations (intra-observer) or mean of three observers (inter-observer and inter-method) served as ground truth. The boxes indicate the IQR, the line within stands for the median and the whiskers indicate points < 1.5 IQR from the box. ‘+’ represents outliers. IQR: interquartile range, between first and third quartile. GT: ground truth.

The median intra-observer measurement differences with the ground truth varied between 0.16 mm (fML) and 0.59 mm (AP LTP). Maximal intra-observer measurement differences ranged between 0.78 mm (AP MFC) and 2.76 mm (AP LTP). The median inter-observer differences ranged between 0.13 mm (AP MFC) and 1.06 mm (AP MTP), whereas maximal differences ranged between 0.83 mm (AP MFC) and 3.02 mm (AP LTP). The mean (and standard deviation) over all inter-observer differences was 0.56 (±0.55) mm. The spread of measurement intra-observer (red) and inter-observer (green) differences is plotted in box-and-whisker plots in Figure 5. Intra-class correlation coefficients for intra- and inter-observer errors were between 0.796 and 1. These are summarized in Table 3.

### 4.2. Validation Study for Automated Morphometric Analysis

#### 4.2.1. Automated Landmark Validation

**Figure 6:**
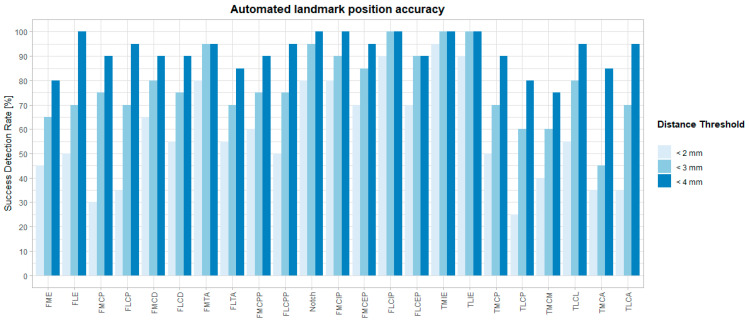
Success detection rates in % per landmark within predefined tolerance [mm].

The median difference from the ground truth landmark position ranged between 0.77 mm (TMIE) and 3.13 mm (TLPA). The maximal inter-method difference varies between 2.02 mm (FLCIP) and 6.99 mm (TPM). For all landmarks, box-and-whisker diagrams are plotted in Figure 4 (blue), and descriptive statistics are in Table 4. Success detection rates at different accuracy thresholds are plotted in Figure 6. For all landmarks, at least 75% of the automated landmarks were placed within 4 mm of the expert mean landmark. On average, 78% and 92% of the landmarks were placed automatically within 3 mm and 4 mm of the expert mean landmark, respectively.

#### 4.2.2. Automated Measurement Validation

**Figure 7:**
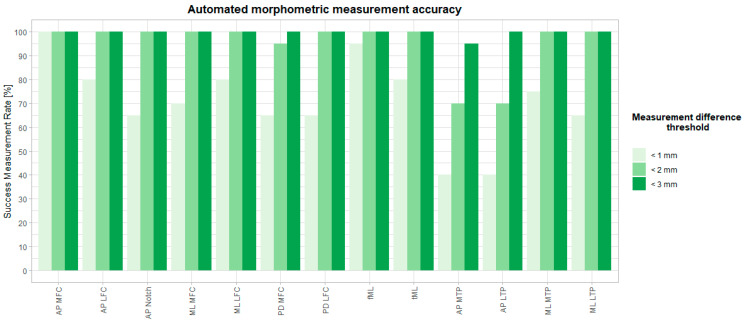
Success measurement rates in % per morphometric measurement within predefined tolerance [mm].

The median difference between the automated and manual methods was between 0.33 mm (AP MFC) and 1.72 mm (AP MTP). Maximal differences ranged between 0.74 mm (AP MFC) and 2.85 mm (AP MTP). A detailed overview per measurement is visualized as a box-and-whiskers diagram in Figure 5, and descriptive statistics are in Table 5. Intraclass correlation coefficients varied between 0.938 and 0.999 and are reported in Table 6. Success measurement rates are plotted in Figure 7 for different accuracy thresholds. For all but two measurements (AP MTP and AP LTP), at least 90% of the automated measurements were less than 2 mm different from the expert mean measurements. On average, over all measurements, 71% and 95% of all measurements had a difference below 1 mm and 2 mm with the expert mean measurement, respectively. Finally, Bland–Altman diagrams (Figure S1 in Supplementary Materials) showed similarly distributed errors over the full measurement range.

### 4.3. Time Consumption

The manual method takes 15–30 min per case for extracting all anatomical landmark coordinates from 3D bone and cartilage shapes. The time varied based on observer experience and image resolution. For one observer and five subject cases, the mean (and standard deviation) of the required time for manual landmarking was 18.6 ± 1.8 min per knee.

The automated method needed ca. seven minutes of computing time per knee on a desktop workstation (Intel i9-9900K with 32 GB of RAM). This can be split up into the following steps: approx. six minutes for femur and tibia registration, less than one second for initial landmark retrieval and a couple of seconds for landmark optimization.

## 5. Discussion

This study successfully validated our suggested approach for automated anatomical landmarking in 3D morphometric analysis of the distal femur and proximal tibia bones. On average, automated landmarks were placed 2.05 mm from the expert mean landmarks, in comparison to a manual inter-observer variability of 1.53 mm. The derived measurements showed a mean absolute difference of 0.78 mm with the expert mean measurements, whereas the mean inter-observer difference was 0.56 mm. Reliability was proven to be excellent for 3D morphometric measurement of the distal femur and proximal tibia, with an ICC (manual versus automated) ranging between 0.926 and 1. Most importantly, the automated landmark extraction algorithm significantly accelerates the process, decreasing manual labor from approximately half an hour of manual work to a mere seven minutes of operator-independent computing time.

Our manual landmark annotations are in line with reported accuracies for the same anatomy. Victor et al. [7] previously reported mean intra-observer differences between 0.41 mm and 1.4 mm and mean inter-observer accuracies between 0.66 mm and 3.5 mm for a highly overlapping set of anatomical landmarks. It should be noted that their 3D bone models were CT-derived (1.25 mm axial slice thickness) and thus potentially had a finer mesh resolution. Van der Merwe et al. [6] obtained mean intra- and inter-observer accuracies in ranges between 0.34 mm and 1.7 mm (intra) and between 0.08 mm and 1.91 mm (inter) on MRI-derived bone models (research scan protocol, 1.5 mm slice thickness, average in-plane resolution of 0.4 mm) of distal femur and proximal tibia.

Recently, a method for the assessment of the full lower limb alignment was proposed and validated on CT-derived 3D bone models of the femur and tibia [20]. Based on a similar technology and applied to a largely overlapping set of landmarks, the authors were able to extract all landmarks with a mean absolute difference of 2.17 mm compared to the manual method. Primarily using identical anatomical landmarks, our results are in line with their reported accuracies between repeated manual annotations and inter-method (manual versus automated) accuracy.

Several factors may impact the accuracy and success rate of automated landmarking. First and foremost, the quality of the expert landmarks plays a pivotal role in establishing the ground truth. Given the limited number of experts and significant inter-observer variability for certain landmarks, it can be argued if the expert mean landmarks truly serve as a robust ground truth [10]. Interobserver dispersion of the landmarks is likely influenced by the difficulty of manually locating the landmark [21] and variations in training or background among observers [22]. Furthermore, keeping in mind the anatomical meaning of certain landmarks (e.g., FME or FLE) as origin or insertion points of a ligament or tendon, multiple candidate landmark points can be considered equally correct, as no ligament or tendon is only attached to the bone by one single fiber. Thus, annotating landmarks is not a trivial task, as it is a simplification of a more complex anatomical reality. To warrant an optimal quality of the expert mean landmarks in the current study, the landmarks were annotated by three independent experts instead of one single observer.

Secondly, the automated landmarking accuracy is related to the mesh resolution of the registered surfaces and the template mesh, as the candidate landmark positions are limited to the vertices of the registered surfaces. A higher resolution (more vertices) in the template mesh enhances accuracy but increases computing time for 3D surface registration. In addition, the resolution and slice thickness of the source imaging also play a role. Subtle ridges, such as the anterior border of the medial and lateral tibial plateau cartilage, might be more or less pronounced. Since our automated approach relies solely on the 3D models derived from thick-slice MRI scans, the TMCA and TLCA landmarks were more challenging to detect, resulting in larger AP TPM and AP TPL differences from the ground truth measurements.

**Figure 8:**
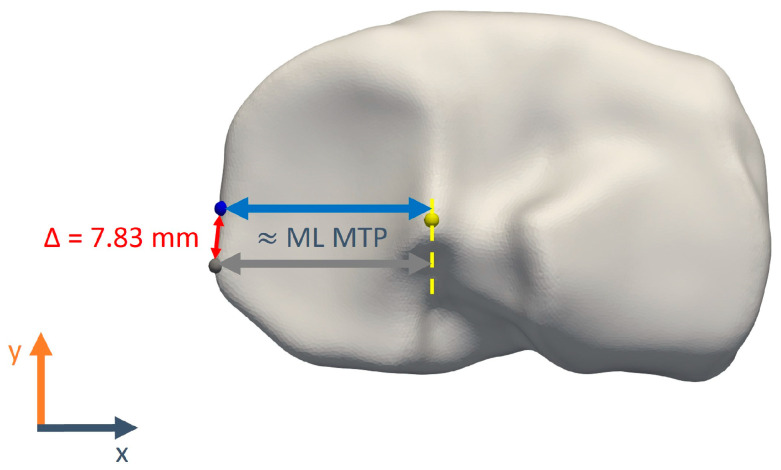
Exaggerated representation of a potential discrepancy between landmark position difference and corresponding measurements difference. Both blue and grey TMCM landmarks conform to the landmark definition, yet there is a 7.83 mm gap between them. In contrast, the calculated medial tibial plateau width only differs by 0.49 mm.

Finally, the surface registration algorithm is prone to the introduction of minor tangential translations of vertices over the 3D surface in nearly flat regions. This is reflected in landmark positioning errors, which do not contribute directly to larger measurement errors (as illustrated in Figure 8). A similar effect was also observed for the manual landmarking: while, e.g., the posterior points on the tibia plateau showed inter-observer differences up to 9.7 mm, the maximal anteroposterior size inter-observer differences were maximally only ca. 3.5 mm. It was indeed verified that the largest part of the TMCP and TLCP position differences is in the mediolateral and proximodistal coordinates.

The main strength of our automated landmark tool is the extensive standardization of the complete process, thereby ruling out any intra- or inter-observer variation and making the time-consuming human input obsolete. The marginal operator time was reduced from up to 30 min per knee to zero after initialization of the landmarks of interest on the template shape. Combining a template-based method with some domain knowledge of the anatomy, a limited amount of training data were proven to be sufficient. Our method does not require huge datasets for training, as is the case with deep learning approaches. While deep learning methods might be able to achieve higher accuracy, the training of these neural networks is slower and requires much more training data. Measuring distances and angles based on well-defined landmarks is probably the most straightforward and understandable method to analyze simple bone and cartilage shape variations. The clinical relevance of this type of bone shape analysis already lies within implant design and patient-specific pre-operative planning [23].

Using the 3D bone and cartilage models instead of the raw images is a potential limitation to evaluating the real-world clinical applicability of this method. Indeed, the focus of this study was on the landmarking process rather than the segmentation of the raw images. Using pre-operative MRI scans of the knee from routine diagnostic procedures for meniscus lesions from different clinical centers does ensure the robustness and generalization abilities of our method. Additionally, considering the data-driven nature of our method, a larger training dataset could be beneficial for automatic landmarking accuracy. However, similar validation studies report acceptable to excellent results based on similar sample sizes [20,24,25,26].

The image quality of the clinical MRI scans (large slice thickness) could be considered a challenge for 3D bone and cartilage segmentation. Iterative verification and finetuning of the 3D models over three mutually perpendicular views were required to result in a manual segmentation accuracy of at least 1 mm [2]. Undoubtedly, ongoing advances in isotropic high-resolution MRI protocols [27] will provide higher-quality 3D models with more fine details (ridges and indents). This will facilitate both manual landmark annotation (a smaller region to focus on while searching the landmark) and automated landmarking (less prone to sliding surface errors during elastic deformation in the registration process).

Future potential improvements include automating the segmentation process from raw images to reduce even further observer-related variability and human processing time. Furthermore, the surface registration algorithm could be accelerated by implementing multithread computing, but this was not the scope of this study. Surface registration remains the main time-consuming factor in the automated method and always entails a trade-off between accuracy and time consumption. A further potential enhancement is the introduction of Mean Value Coordinates [28], a generalization of barycentric coordinates. It overcomes the limitation of the registered mesh vertices being the only candidate landmark positions, allowing a lower template mesh resolution for a similar achievable landmarking accuracy.

In conclusion, considering the substantial variability among observers in the manual method, there is a clear need for an objective, operator-independent, and efficient approach to identifying anatomical landmarks. Our automated method demonstrated excellent accuracy and reliability for both landmark positioning and morphometric measurements. Moreover, this high level of automation will lead to a faster, scalable and human operator-independent morphometric analysis of the knee. Potential applications include optimized orthopedic implant designs, patient-specific treatment tailoring and large-scale morphometric risk factor analysis in different pathologies.

## Figures and Tables

**Table 1:** Landmark definitions.

Acronym	Landmark	Definition
FME	Femoral medial epicondyle	The most anterior and distal osseous prominence over the medial aspect of the 3D distal femur.
FLE	Femoral lateral epicondyle	The most anterior and distal osseous prominence over the lateral aspect of the 3D distal femur.
FMCP	Femoral medial condyle posterior	The most posterior point of the medial condyle on the 3D femur.
FLCP	Femoral lateral condyle posterior	The most posterior point of the lateral condyle on the 3D femur.
FMCD	Femoral medial condyle distal	The most distal point on the medial condyle on the 3D femur.
FLCD	Femoral lateral condyle distal	The most distal point on the lateral femoral condyle on the 3D femur.
FMTA	Femoral medial trochlea anterior	The most anterior point of the medial trochlea on the 3D femur.
FLTA	Femoral lateral trochlea anterior	The most anterior point of the lateral trochlea on the 3D femur.
FMCPP	Femoral medial condyle posterior proximal	The most proximal point of the cartilage at the posterior medial condyle on the 3D femur. Verified on a sagittal MRI view.
FLCPP	Femoral lateral condyle posterior proximal	The most proximal point of the cartilage at the posterior lateral condyle on the 3D femur. Verified on a sagittal MRI view.
Notch	Femoral notch	The most anterior point in the middle of the femoral notch on a caudal to cranial view of the 3D femur
FMCIP	Femoral medial condyle internal point	The most lateral point of the cartilage of the medial condyle on a caudal to cranial view of the 3D femur, at the level of one third of the notch depth anteroposteriorly. Verified on a coronal MRI view.
FMCEP	Femoral medial condyle external point	The most medial point of the cartilage of the medial condyle on a caudal to cranial view of the 3D femur, at the level of one third of the notch depth anteroposteriorly. Verified on a coronal MRI view.
FLCIP	Femoral lateral condyle internal point	The most medial point of the cartilage of the lateral condyle on a caudal to cranial view of the 3D femur, at the level of one third of the notch depth anteroposteriorly. Verified on a coronal MRI view.
FLCEP	Femoral lateral condyle external point	The most lateral point of the cartilage of the lateral condyle on a caudal to cranial view of the 3D femur, at the level of one third of the notch depth anteroposteriorly. Verified on a coronal MRI view.
TMIE	Tibial medial intercondylar eminence	The most proximal or highest point of the medial intercondylar eminence.
TLIE	Tibial lateral intercondylar eminence	The most proximal or highest point of the lateral intercondylar eminence.
TMCP	Tibial medial condyle posterior	The most posterior and lateral point of the medial compartment on the 3D tibia. Verified on a sagittal MRI view.
TLCP	Tibial lateral condyle posterior	The most posterior and medial point of the lateral compartment on the 3D tibia. Verified on a sagittal MRI view.
TMCM	Tibial medial condyle medial	The most medial point of the tibial plateau on the 3D tibia, axially aligned following the posterior condylar line of the corresponding femur.
TLCL	Tibial lateral condyle lateral	The most lateral point of the tibial plateau on the 3D tibia, axially aligned following the posterior condylar line of the corresponding femur.
TMCA	Tibial medial condyle anterior	The most anterior point on the cartilage of the medial tibial plateau (on a sagittal MRI view)
TLCA	Tibial lateral condyle anterior	The most anterior point on the cartilage of the lateral tibial plateau (on a sagittal MRI view)

**Table 2:** Morphometric measurement definitions.

Measurement Abbreviation	Measurement Definition	Between Landmarks	Measurement Projection Axis
AP MFC	Anteroposterior size of the medial femoral condyle	FMCP, FMTA	y (AP)
AP LFC	Anteroposterior size of the lateral femoral condyle	FLCP, FLTA	y (AP)
AP notch	Anteroposterior size of the femoral notch	FMCP, Notch (FLCP, Notch)	y (AP)
fML	Mediolateral size of the distal femur	FME, FLE	x (ML)
ML MFC	Mediolateral size of the medial femoral condyle	FMCIP, FMCEP	x (ML)
ML LFC	Mediolateral size of the lateral femoral condyle	FLCIP, FLCEP	x (ML)
ML notch	Mediolateral size of the femoral notch	FMCIP, FLCIP	x (ML)
PCL	Posterior condylar line	FMCP, FLCP	x (ML)
PD MFC	Proximodistal size of the medial femoral condyle	FMCPP, FMCD	z (PD)
PD LFC	Proximodistal size of the lateral femoral condyle	FLCPP, FLCD	z (PD)
AP MTP	Anteroposterior size of the medial tibial plateau	TMCP, TMCA	y (AP)
AP LTP	Anteroposterior size of the lateral tibial plateau	TLCP, TLCA	y (AP)
tML	Mediolateral size of the tibial plateau	TMCM, TLCL	x (ML)
ML MTP	Mediolateral size of the medial tibial plateau	TMCM, TMIE	x (ML)
ML LTP	Mediolateral size of the lateral tibial plateau	TLCL, TLIE	x (ML)

**Table 3:** Intraclass correlation coefficients for reliability analysis of the measurements.

Measurement	ICC_intra_	ICC_inter_	Measurement	ICC_intra_	ICC_inter_
AP MFC	1	1	AP MTP	1	0.999
AP LFC	1	1	AP LTP	0.999	0.998
ML MFC	0.937	0.796	ML MTP	0.953	0.94
ML LFC	0.985	0.922	ML LTP	0.955	0.935
PD MFC	0.983	0.947	tML	0.986	0.968
PD LFC	0.976	0.958	fML	0.991	0.969
AP Notch	1	1			

**Table 4:** Descriptive statistics for intra-observer, inter-observer and inter-method (manual versus automated) landmark position differences. Only for the intra-observer differences the mean of three observations was used as reference landmark position. Expert mean landmarks were used as reference landmark positions to assess inter-observer and inter-method landmark differences. SD: standard deviation.

Landmark Acronym	Mean (SD) Intra-Observer	Mean (SD) Inter-Observer	Mean (SD) Inter-Method	Landmark Acronym	Mean (SD) Intra-Observer	Mean (SD) Inter-Observer	Mean (SD) Inter-Method
FME	1.43 (1.26)	2.21 (1.59)	2.59 (1.48)	FMCIP	0.69 (0.35)	1.26 (0.78)	1.56 (0.88)
FLE	1.52 (1.45)	1.47 (1.28)	2.31 (1.07)	FMCEP	1.05 (1.18)	1.74 (1.03)	1.70 (1.22)
FMCP	1.52 (0.91)	1.40 (0.77)	2.51 (1.23)	FLCIP	0.54 (0.32)	1.13 (0.84)	1.08 (0.70)
FLCP	1.59 (1.02)	1.33 (0.95)	2.54 (1.28)	FLCEP	0.70 (0.50)	2.08 (1.59)	1.93 (1.25)
FMCD	1.56 (1.33)	1.56 (1.02)	1.98 (1.26)	TMIE	0.57 (0.49)	0.62 (0.30)	0.90 (0.53)
FLCD	1.14 (0.87)	1.85 (1.30)	2.06 (1.54)	TLIE	0.41 (0.25)	0.58 (0.30)	1.05 (0.68)
FMTA	0.91 (0.65)	1.21 (0.83)	1.47 (0.91)	TMCP	0.99 (0.60)	2.13 (1.67)	2.29 (1.33)
FLTA	1.23 (0.90)	1.44 (0.98)	2.25 (1.20)	TLCP	1.38 (0.94)	2.58 (1.76)	2.86 (1.45)
FMCPP	0.85 (0.56)	1.55 (0.96)	2.25 (1.34)	TMCM	1.19 (1.01)	1.87 (1.72)	2.75 (1.88)
FLCPP	0.78 (0.49)	1.16 (0.65)	2.14 (0.99)	TLCL	1.12 (0.76)	1.23 (0.61)	2.13 (1.18)
Notch	0.69 (0.47)	0.82 (0.46)	1.51 (0.66)	TMCA	1.64 (0.98)	2.31 (1.34)	2.84 (1.39)
All landmarks	1.07 (0.92)	1.53 (1.22)	2.05 (1.30)	TLCA	1.21 (0.66)	1.63 (1.00)	2.41 (1.15)

**Table 5:** Descriptive statistics for measurement differences intra-observer, inter-observer, and inter-method (manual versus automated). Only for the intra-observer differences the mean of three observations was used as reference landmark measurement. Expert mean landmarks were used to calculate the reference measurements to assess inter-observer and inter-method landmark differences. SD: standard deviation.

Measurement	Mean (SD) Intra-Observer	Mean (SD) Inter-Observer	Mean (SD) Inter-Method	Measurement	Mean (SD) Intra-Observer	Mean (SD) Inter-Observer	Mean (SD) Inter-Method
AP MFC	0.21 (0.16)	0.18 (0.17)	0.54 (0.21)	AP MTP	0.50 (0.33)	1.19 (0.77)	1.39 (0.91)
AP LFC	0.28 (0.22)	0.23 (0.22)	0.67 (0.36)	AP LTP	0.74 (0.64)	1.11 (0.81)	1.46 (0.92)
ML MFC	0.33 (0.28)	0.61 (0.42)	0.66 (0.41)	ML MTP	0.30 (0.27)	0.35 (0.30)	0.65 (0.41)
ML LFC	0.24 (0.22)	0.62 (0.43)	0.69 (0.45)	ML LTP	0.42 (0.40)	0.50 (0.42)	0.75 (0.57)
PD MFC	0.29 (0.22)	0.45 (0.45)	0.80 (0.51)	tML	0.43 (0.38)	0.70 (0.47)	0.57 (0.45)
PD LFC	0.33 (0.25)	0.43 (0.32)	0.85 (0.51)	fML	0.30 (0.35)	0.63 (0.60)	0.51 (0.28)
AP Notch	0.40 (0.31)	0.46 (0.31)	0.69 (0.53)	All measurements	0.36 (0.35)	0.56 (0.55)	0.78 (0.60)

**Table 6:** Intraclass correlation coefficients (manual versus automated) for reliability analysis of the measurements.

Measurement	ICC	Measurement	ICC	Measurement	ICC
AP MFC	1	AP Notch	1	AP MTP	0.999
AP LFC	1	PD MFC	0.961	AP LTP	0.999
ML MFC	0.926	PD LFC	0.951	ML MTP	0.944
ML LFC	0.966	fML	0.995	ML LTP	0.956
		tML	0.993		

## Data Availability

The data presented in this study are available on request from the corresponding author.

## Supplementary Materials

The following supporting information can be downloaded at: https://www.mdpi.com/article/10.3390/jimaging10040090/s1, Figure S1: Bland–Altman inter-method agreement plots for all measurements: differences between the manual and automated measurement (mm) are plotted in function of the average of the manual and automated measurement; Table S1: Number of outliers in landmark positioning differences per knee scan for each of the described experiments: intra-observer, inter-observer and manual versus automated, Table S2: Number of outliers in measurement differences per knee scan for each of the described experiments: intra-observer, inter-observer and manual versus automated.
