# Supplementary material for: Automated Landmark Annotation for Morphometric Analysis of Distal Femur and Proximal Tibia
Source: J Imaging. 2024 Apr 11;10(4):90. doi: 10.3390/jimaging10040090 (PMC11051533; doi:10.3390/jimaging10040090)
Supplement: Supplementary file 1 [file jimaging-10-00090-s001.zip › jimaging-2905857-supplementary.pdf]

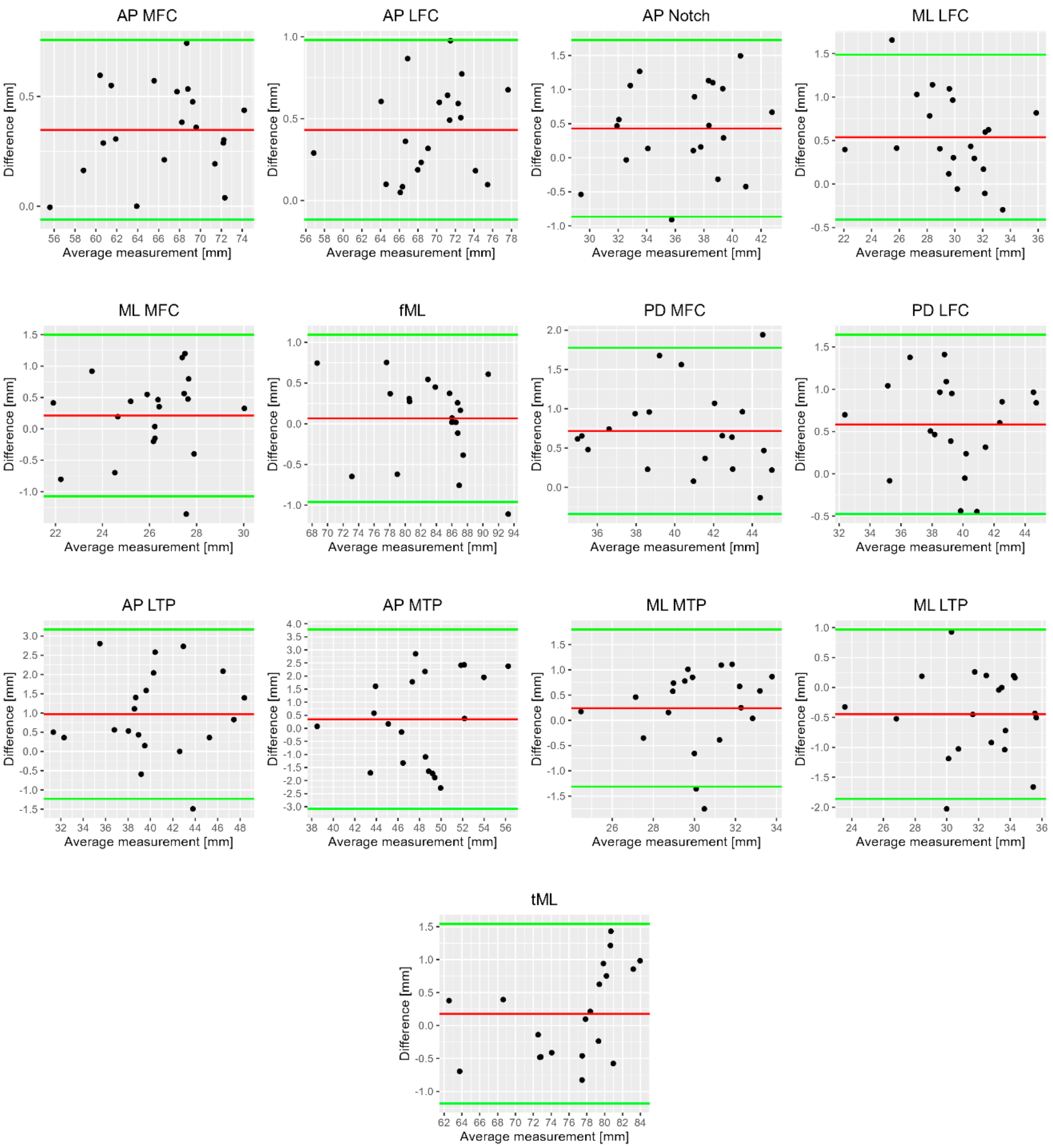

Figure S1: Bland Altman inter-method agreement plots for all measurements: differences between the manual and automated measurement (mm) are plotted in function of the average of the manual and automated measurement.

Landmark positioning outliers

Table S1: Number of outliers in landmark positioning differences per knee scan for each of the described experiments: intra-observer, inter-observer and manual versus automated.

Scan	Number of outliers (intra-observer)	Number of outliers (inter-observer)	Number of outliers (manual versus automated
#1	0	5	0
#2	2	1	0
#3	6	5	2
#4	3	2	1
#5	3	2	0
#6	0	5	4
#7	1	2	1
#8	0	3	0
#9	5	1	0
#10	8	0	0
#11	6	1	1
#12	2	3	0
#13	3	1	0
#14	0	0	0
#15	0	6	0
#16	1	2	0
#17	6	5	2
#18	9	2	0
#19	3	2	2
#20	3	0	0
**Total**	**61**	**48**	**13**

Measurement outliers

Table S2: Number of outliers in measurement differences per knee scan for each of the described experiments: intra-observer, inter-observer and manual versus automated.

Scan	Number of outliers (intra-observer)	Number of outliers (inter-observer)	Number of outliers (manual versus automated
#1	1	7	0
#2	0	0	0
#3	2	0	0
#4	1	0	0
#5	1	0	0
#6	0	1	0
#7	0	2	0
#8	0	2	0
#9	2	0	0
#10	5	1	1
#11	4	0	0
#12	1	1	0
#13	4	0	1
#14	3	0	0
#15	1	1	0
#16	2	1	0
#17	4	6	1
#18	3	2	0
#19	0	0	0
#20	3	0	0
**Total**	**37**	**24**	**3**
